# Faecal tumour pyruvate kinase M2: not a good marker for the detection of colorectal adenomas

**DOI:** 10.1038/sj.bjc.6604656

**Published:** 2008-09-30

**Authors:** Y M Shastri, J M Stein

**Affiliations:** 1 Department of Gastroenterology and clinical nutrition, St Elisabethen Hospital, Katharina Kasper Kliniken, Goethe-University, Frankfurt am Main, Germany

**Sir,**

We read with great interest the article by Haug *et al* (2008) and would like to complement them for such interesting clinical work. Their study further supports the previous smaller studies in patients with more-than-average risk of harbouring colorectal cancer (CRC) (Shastri *et al*, 2006, 2008), in which the suboptimal performances of faecal pyruvate kinase M2 (M2-PK) for detecting the precursors for CRC (the colorectal adenomas) have already been demonstrated. However, we have certain comments to make about this study:

1. As per the study design, they performed CRC screening in all the asymptomatic patients older than 55 years (as per the screening guidelines in Germany). But in Table 1, the first two groups of patients were in the range of 30–49 and 50–59 years. How can this be explained when only patients older than 55 years were included in the study?
2. In this study, all those average-risk patients scheduled for colonoscopic CRC screening were included and were requested to provide stool samples for performing faecal M2-PK. They included close to 1100 patients undergoing screening colonoscopy in 20 participating centres over a period of nearly 2 years. Thus on an average 28 colonoscopies/year (about two screening colonoscopies/month) were performed in each centre for this study. Are these not a small number of CRC screenees undergoing colonoscopies in these centres!
3. In the Materials and Methods section it is mentioned that ‘Patients with insufficient knowledge of German language were excluded’. What could have been the reason for this? As there is not much history taking required when suitable asymptomatic screenees undergo CRC screening?

We agree with the authors of the study (Haug *et al*, 2008) that faecal M2-PK has poor test characteristics (especially low sensitivity and specificity) in distinguishing between patients having colorectal adenomas or no clinical findings at colonoscopy. This has also been reported in a previous prospective study (Mulder *et al*, 2007). This knowledge further consolidates the case for not recommending faecal M2-PK as a biomarker for CRC screening, as detecting and removing the precursors of CRC is one of the major aims of any CRC screening modality.
